# Measuring high-sensitivity cardiac troponin T blood concentration in population surveys

**DOI:** 10.1371/journal.pone.0171242

**Published:** 2017-01-31

**Authors:** Antonio Ivan Lazzarino, Jennifer S. Mindell

**Affiliations:** 1 London School of Hygiene & Tropical Medicine, London, United Kingdom; 2 Department of Epidemiology and Public Health, UCL, London, United Kingdom; The University of Tokyo, JAPAN

## Abstract

**Introduction:**

The blood test for high-sensitivity cardiac troponin T (HS-CTnT) has been proposed as a marker of cardiovascular risk in the general population, as it is associated with subsequent incidence of cardiovascular events and mortality. We aimed at evaluating the feasibility of HS-CTnT testing within large nationally-representative population surveys in which blood samples are collected during household visits, shipped using the standard civil postal service, and then frozen for subsequent analyses.

**Methods:**

The Health Survey for England (HSE) consists of a series of annual surveys beginning in 1991. It is designed to provide regular information on various aspects of the nation’s health and risk factors. We measured HS-CTnT in the blood of 200 people from the HSE 2016 wave, then froze and stored their blood samples at -40°C for 5–10 weeks, and then thawed and retested them to appreciate the extent of within-person agreement or test-retest reliability of the two measurements.

**Results:**

The Cronbach's Alpha (Scale Reliability Coefficient) and the Interclass Correlation Coefficient (two-way mixed-effects model for consistency of agreement at individual level) were 0.97 (95%CI = 0.96–0.99) and 0.95 (95%CI = 0.94–0.96) respectively. The time delay from blood withdrawal to analysis and storage (1–4 days) did not affect the results, nor did the freezing time before the retest (5–10 weeks).

**Conclusion:**

The measurement of HS-CTnT plasma concentration within large nationally-representative surveys such as the Health Survey for England is feasible.

## Introduction

Cardiac troponin is a protein operating within the heart muscle cell. During a heart attack (acute myocardial infarction) these cells rupture and troponin spills out into the main blood stream; troponin can be detected in the peripheral venous blood as a marker of cardiac damage. Cardiac troponin is the gold standard biomarker for diagnosing patients with heart attack in acute clinical settings.[1] High-sensitivity assays have recently been developed and the troponin subunit T (HS-CTnT) has shown the highest epidemiological sensitivity.[2,3] With the advent of high-sensitivity assays, troponin positivity has become a common finding even in individuals free from cardiovascular symptoms and even outside clinical settings, possibly due to leakage through the membranes of partially-damaged heart cells or due to some other unknown mechanism.[4] In healthy people, HS-CTnT positivity is associated with greater subsequent incidence of cardiovascular disease and mortality and can therefore be considered as the most proximal sentinel marker of heart disease and an index of cardiovascular health.[5,6]

For these reasons, the testing for plasma HS-CTnT has been proposed as a marker of cardiovascular risk in the general population and some large studies have already been conducted using that marker at population level.[6,7] HS-CTnT elevation in healthy people has also been studied in association with socioeconomic conditions using stored blood samples.[8–11]

However, the HS-CTnT assay was designed for hospital use and may be inadequate for population surveys, as in hospitals the test is carried out in emergency settings and the blood samples are analysed immediately after the blood withdrawal, whereas in population surveys the blood is not analysed immediately and it is often frozen for subsequent analyses.

We aimed at evaluating the feasibility of HS-CTnT testing within nationally-representative population surveys such as the Health Survey for England, in which blood samples are collected during household visits, shipped using the standard civil postal service, centrifuged and then the serum is frozen for subsequent analyses.

## Methods

### Study design

The Health Survey for England (HSE) is a nationally representative, general population-based study that recruits individuals living in private households in England using stratified random sampling.[12] The HSE consists of a series of annual surveys beginning in 1991 and it is designed to provide regular information on various aspects of the nation’s health and risk factors.[12,13] All HSE years have the same core measurements but each survey year also has special focus topics that change from one year to another (e.g. cardiovascular disease [CVD], accidents, respiratory conditions, kidney disease, etc.). The HSE blood samples are collected by trained nurses during household visits, and then posted immediately using special preformed, thin, absorbent packaging within printed leakproof bags to the laboratory for biochemical analysis using first class Royal Mail. Once arrived at the laboratory, blood samples from the plain vacutainer tube (no preservative or anticoagulant) are centrifuged and the serum is analysed; residual serum from participants who had given written consent is then stored at -40°C.[12] Ethical approval for HSE 2016–2019 was obtained from the East Midlands Research Ethics Committee (reference no. 15/EM/0254). Study participants gave fully-informed written consent.

### Data collection

We analysed HS-CTnT in the blood of 200 people from the 2016 HSE wave. The collection of blood samples took place between 28 June and 4 August 2016. The average temperature in that period of time in England was 16.7°C (Min 12.3 –Max 21.1). The serum samples were frozen and stored for 5–10 weeks, and then thawed and retested to appreciate the extent of within-person agreement or test-retest reliability of the two measurements. There is a time delay from the blood draw to the arrival of the samples at the laboratory, and in order to check if the delay affects the test results or not, we set out to measure HS-CTnT on 50 random samples in each ‘delay category’ (day 1, 2, 3, and 4). We also checked if freezing time affects the test results or not by retesting a third of each delay category after 5 to 7 weeks, 8–9 weeks, and at 10 weeks.

### Data analysis

We carried out statistical analyses using several approaches. We compared the test results between the original and frozen samples at individual level using the Cronbach's Alpha (Scale Reliability Coefficient) and the Interclass Correlation Coefficient (two-way mixed-effects model for consistency of agreement at individual level).[14] The confidence interval for the Cronbach’s Alpha was calculated using bootstrapping (1,000 replications).

We then recoded the crude values to label the study participants as being at low or high risk (binary variable). We carried out this kind of analysis three times, each time applying a different cut-off value: first at 3 ng/L, which is the lower limit of detection of the assay; then at 10ng/L, which corresponds to the 90^th^ percentile in our dataset; and finally at 14 ng/L, which corresponds to the 95^th^ percentile in our dataset and is also a recognised threshold to mark elevated levels.[1,5,7] We then used the Kappa statistics to compare the extent of agreement between the original and frozen measures for each of the three thresholds.

As a sensitivity analysis, HS-CTnT was log-transformed (before the transformation, zero values were recoded to 1.5 ng/L, which equals the half of the lowest detection limit of the assay). All computations were made using Stata v.14.2.

## Results

There was only one missing value due to insufficient blood sample and the final analytic sample therefore included 199 observations. HS-CTnT was detectable (≥3 ng/L) in 29.0% of the original samples (95%CI = 22.9–36.0) and in 28.1% of the frozen sample (95%CI = 22.0–34.9). Table 1 shows a comparison of HS-CTnT findings between the original and frozen blood samples.

**Table 1:** High-sensitivity cardiac troponin T measurements for 199 individuals drawn from the Health Survey for England 2016.

HS-CTnT		Type of blood sample	
		Original	Frozen
Detectable	n (%)	58 (29.1)	56 (28.1)
Elevated	n (%)	11 (5.5)	10 (5.0)
Minimum value if detectable	ng/L	5	5
Maximum value if detectable	ng/L	38	27
Arithmetic mean ± SD if detectable	ng/L	10.4 ±5.6	9.8 ±4.5
Geometric mean ± SD if detectable	ng/L	9.4 ±1.5	9.0 ±1.5
Median ± IQR if detectable	ng/L	9.5 ±5.0	9.0 ±6.0
90th Percentile for the whole sample	ng/L	10.0	10.0
95th Percentile for the whole sample	ng/L	14.0	14.0
99th Percentile for the whole sample	ng/L	26.0	25.0

Detectable concentrations are those ≥3 ng/L. Elevated concentrations are those ≥14 ng/L. SD = standard deviation. IQR = interquartile range.

The test-retest reliability between the original and frozen sample gave the following results: Cronbach's alpha = 0.97 (95%CI = 0.96–0.99); ICC = 0.95 (95%CI = 0.94–0.96).

Table 2 shows the Cronbach's alpha and the ICC across categories of time delay (from blood withdrawal to analysis) and of freezing time (from start of storage to thaw and retest). The coefficients from these analyses were also close to 1 (perfect consistency).

**Table 2:** Cronbach's Alpha (Scale Reliability Coefficient) and the Interclass Correlation Coefficient (two-way mixed-effects model for consistency of agreement at individual level) for the test-retest reliability between original and frozen samples for 199 individuals drawn from the Health Survey for England, United Kingdom, in year 2016.

Subgroup	N	Type of comparison	
		Cronbach’s Alpha (95%CI)	ICC (95%CI)
Delay days = 1	50	0.98 (0.96 to 1.00)	0.96 (0.93 to 0.98)
Delay days = 2	50	0.96 (0.92 to 1.01)	0.93 (0.88 to 0.96)
Delay days = 3	50	0.92 (0.84 to 1.01)	0.85 (0.75 to 0.91)
Delay days = 4	49	0.99 (0.96 to 1.01)	0.97 (0.95 to 0.98)
Freezing weeks <8	63	0.98 (0.97 to 1.00)	0.97 (0.95 to 0.98)
Freezing weeks = 8–9	77	0.98 (0.94 to 1.01)	0.96 (0.93 to 0.97)
Freezing weeks = 10	59	0.97 (0.94 to 0.99)	0.93 (0.89 to 0.96)
**Whole sample**	**199**	**0.97 (0.96 to 0.99)**	**0.95 (0.94 to 0.96)**

Delay days = number of days from blood withdrawal to analysis and storage of original blood samples. Freezing weeks = number of weeks during which samples were kept frozen at −40°C before thaw and retest.

Table 3 shows the results of the kappa statistics for the three cut-off points. The most efficient cut-off point was 10 ng/L (K = 0.91, 95%CI = 0.83–1.00), although the other cut-off points also showed evidence of very good agreement between original and frozen samples.

**Table 3:** Kappa statistics for the agreement between original and frozen HS-CTnT samples in flagging high-risk individuals using different cut-off points, for 199 participants drawn from the Health Survey for England, United Kingdom, in year 2016.

Cut-off	Kappa (95%CI)
≥3 ng/L	0.88 (0.80 to 0.95)
≥10 ng/L	0.91 (0.83 to 1.00)
≥14 ng/L	0.85 (0.68 to 1.00)

## Discussion

Our results indicate that measuring HS-CTnT within population studies such as the Health Survey for England is feasible, and that the analysis of blood samples stored for several weeks at -40°C gives consistent results compared to the analysis of original (although delayed) samples, with statistical results close to perfection.

In the HSE, nurses collect blood samples during household visits and then ship them using the standard civil postal service. Therefore, samples may stay at room/external temperature for 1–4 days before reaching the laboratory for analysis and storage and this may add inaccuracy to the analyses. However, although HS-CTnT should be analysed immediately after blood withdrawal, there is evidence that HS-CTnT is stable for at least 24 h in whole blood at room temperature.[15] In that study, blood samples were centrifuged after storage at room temperature (20–23°C) for 15 min, 4, 8, and 24 h. All HS-CTnT results in each series were in a range from –3.9% to +2.5% compared with the results at 15 min. This was within what was expected due to assay imprecision. Such stability was consistent across time delays, and there was no trend showing increasing instability with increasing time delay. It is therefore likely that hypothetical enzymatic or non-enzymatic processes that do not affect HS-CTnT detection within 24 h are also not operating in the subsequent hours. Moreover, two more recent studies reported that HS-CTnT testing is not affected by preanalytical variation in type of blood collection tube, prolonged transport, centrifugation time and speed, and storage conditions.[16,17]

However, our results may not be generalizable to countries where the external temperature is much higher than in England, as protein denaturation may happen with the elevated external temperatures that are reached in some countries.

In acute settings, the diagnosis of a heart attack is centred on HS-CTnT test findings above the arbitrary threshold value of 14.0 ng/L,[2] corresponding to a value of HS-CTnT at the 99^th^ percentile that was derived from small studies of presumably healthy individuals, with relatively little phenotypic characterisation, and not representing the English population. There is evidence that the value of cardiac troponin blood concentration at the 99^th^ percentile differs according to demographic and clinical conditions.[2,18] In this study, we found a value of HS-CTnT at the 99^th^ percentile of 25 ng/L. Therefore using 14 ng/dL as a cut-off point to flag people with a heart attack in English hospitals may lead to false-positive diagnoses. However, although the HSE is a nationally-representative survey, we carried out our analyses on a subsample of that survey, and therefore our sample may not be representative of the English population. Variables such as age, gender, location, and other demographic and clinical variables were not available for analysis and we therefore could not study them in relation to HS-CTnT. Furthermore we cannot exclude the possibility that some of our participants had heart disease. Therefore we encourage further research in estimating population percentiles for HS-CTnT, as has also been suggested by a recent NICE guideline (National Institute for Health and Care Excellence).[2]

Now that we have shown that the measurement of HS-CTnT plasma concentration within nationally-representative surveys such as the Health Survey for England is feasible, there may be several possible applications of HS-CTnT surveys. The HS-CTnT percentiles may vary according to clinical and demographic population conditions, and therefore we encourage the development of nationally representative, nation-specific population surveys to calculate nation-specific thresholds of HS-CTnT for the diagnosis of a heart attack within each healthcare system. Another objective could be to map the prevalence of HS-CTnT positivity at regional, national and international level and in population sub-groups, to identify the areas of the world or the population subgroups that are at higher risk of heart disease. The traditional risk factors for CVD that are measured in population surveys are not sufficient to explain the burden of CVD in the world. This is because risk factors such as blood pressure and cholesterol are unable to fully explain the development of atherosclerosis and thrombosis, and atherosclerosis and thrombosis are not the only precursors of CVD.[9] By being a more proximal marker for CVD and an alert signal prior to CVD events, HS-CTnT positivity incorporates and integrates the effects of the other more distal risk factors. HS-CTnT is therefore a good candidate to become a useful marker of cardiovascular health that could be used at a population level.

## Supporting information

S1 File Individual level data. (XLSX)
